# Measurement of spin–orbit torque using field counterbalancing in radial current geometry

**DOI:** 10.1038/s41598-023-46658-z

**Published:** 2023-11-08

**Authors:** Jong Wan Son, Seungmo Yang, Tae-Seong Ju, Chanyong Hwang, Kyoung-Woong Moon

**Affiliations:** Quantum Spin Team, Korea Research Institute of Standards and Science, Daejeon, 34113 Republic of Korea

**Keywords:** Spintronics, Applied physics

## Abstract

Controlling the direction of magnetization with an electric current, rather than a magnetic field, is a powerful technique in spintronics. Spin–orbit torque, which generates an effective magnetic field from the injected current, is a promising method for this purpose. Here we show an approach for quantifying the magnitude of spin–orbit torque from a single magnetic image. To achieve this, we deposited two concentric electrodes on top of the magnetic sample to flow a radial current. By counterbalancing the current effect with an external magnetic field, we can create a stable circular magnetization state. We measure the magnitude of spin–orbit torque from the stable radius, providing a new tool for characterizing spin–orbit torque.

## Introduction

Controlling the magnetization direction of magnetic materials with an electric current has been a research focus for some time, as this technology has numerous practical applications beyond academic interest^1–6^. Ampere’s law of electromagnetism describes the magnetic field generated around a flowing current, which has traditionally been used for technology implementation. However, as devices have become smaller, magnetic field interference and energy efficiency have become limiting factors, necessitating new approaches. One such approach is spin transfer torque, which involves injecting electron spins directly into a magnetic sample to change its magnetization state^7–10^.

Efficient spin transfer effects have been observed in heterostructures consisting of a magnetic material and a heavy metal in contact. When a current passes through the heavy metal, the spin-Hall effect causes spin accumulation on each of its lateral surfaces, resulting in the injection of accumulated spins into the adjacent magnetic material^11–13^. This generates spin torque, known as the spin–orbit torque (SOT)^14–18^. The use of this phenomenon has become increasingly common in spintronics research and applications.

Quantifying the strength of the SOT has become a critical issue in spintronics research, as it is directly related to the efficiency of device operation^19–22^. To this end, various measurement methods have been proposed. One widely used approach is the harmonic Hall voltage measurement, which measures the harmonic signals using a lock-in amplifier on a Hall cross geometry^23–26^. Another established method is the spin torque ferromagnetic resonance (ST-FMR), in which a radio frequency (RF) signal is applied to a waveguide-patterned sample, causing oscillation of the magnetization state via spin torque^27–29^. Resonance curves obtained by changing the magnetic field or RF frequency are analyzed. In addition to electrical methods, the SOT can also be measured optically using the magneto-optical Kerr effect (MOKE) with a focused laser light source^30–32^.

While the methods described thus far assume a uniform magnetization state in the observation area, there is an alternative method that enables the observation of non-uniform magnetization changes. This method involves the use of a microscope to observe the magnetization state of the entire area at once, with a MOKE microscope being a typical example^33–36^. Using this microscope, it is possible to observe the phenomenon in which the entire magnetization state of the sample is pushed in one direction due to the SOT effect^35,37,38^. The larger the SOT, the faster the magnetization state moves. A narrow wire structure often employed to better define the current direction and increase the current density.

This paper proposes an alternative method for measuring SOT based on image observation. Unlike existing methods, this approach involves the use of a non-uniform current, exploiting the point at which the effects of the current and the external magnetic field balance each other. Specifically, the non-uniform current generates SOT distributions of varying intensities at different positions, resulting in no magnetization state change at the point where the effect of SOT is counterbalanced by the external magnetic field. By applying this method, the SOT efficiency can be quantified from a single image.

## Results and discussion

**Figure 1:**
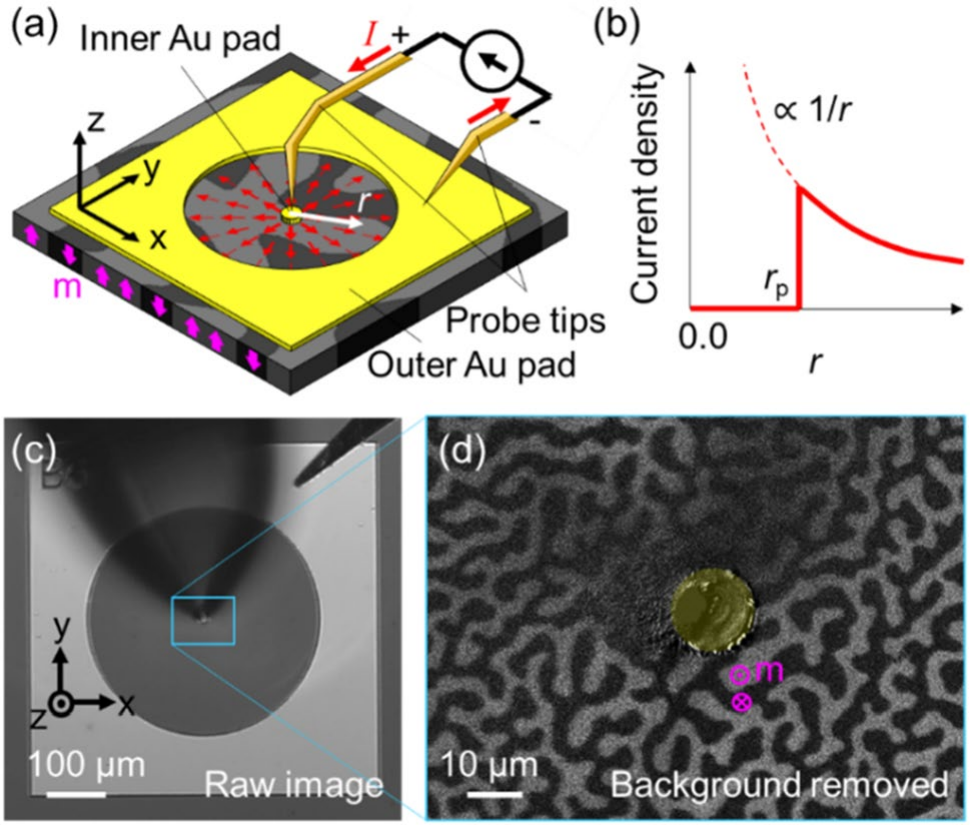
Preparation of pads and contact of vertical probe tip for radial current measurements. (**a**) Schematic of the experimental setup, with red arrows indicating the direction of current flow and pink arrows indicating the direction of magnetization. (**b**) Plot of current density as a function of distance. (**c**) Image of the sample with probe tips in contact. (**d**) Close-up of the magnetization state near the inner pad.

Figure 1a depicts a schematic of the experimental setup. A magnetic film was prepared with a stack sequence of Si/SiO_2_(substrate)/MgO(1 nm)/CoFeB(1.3 nm)/W(3 nm)/TaOx(3 nm) that exhibits perpendicular magnetic anisotropy (PMA), meaning that the magnetization direction (**m**) prefers to align perpendicular to the film plane ($\pm z$ directions, as indicated by pink arrows)^38,39^. Two gold electric pads (thickness 100 nm) were deposited on top of the magnetic film, one with a round perforated shape (outer pad) and the other with a circular disk shape (inner pad, the radius is $r_{{\text{p}}}$) located at the center of the outer pad. The pad structure was designed to allow for a radial current to flow (indicated by red arrows) from the inner pad to the outer pad via probe tips. The current density will have a dependence of $1/r$, where $r$ is the radial distance from the center of the inner pad as shown in Fig. 1b.

The real image of the probe tips in contact with the inner pad is shown in Fig. 1c. The tips were aligned vertically to minimize the Oersted field, which may be asymmetric. Although it was initially a concern that the observation area would be covered by the probe tip, the sample state could be observed except for the area near the inner pad. A trivial magnetization state of the sample at zero magnetic field and zero current with the probe tip in contact is shown in Fig. 1d. Such magnetic states were observed with a MOKE microscope^33^. In order to clearly observe the magnetization state, a background subtraction method was applied to all magnetic images in this paper. The background image was obtained by setting all magnetization directions to a uniform $- z$ direction by applying a sufficiently large magnetic field in the $- z$ direction. The observed magnetization state formed a stripe pattern with alternating $+ z$ and $- z$ magnetization regions of a certain repetition width, which is known as a stripe state^39–41^. The light gray and dark gray areas in Fig. 1d indicate the $+ z$ and $- z$ directions of magnetization, respectively.

**Figure 2:**
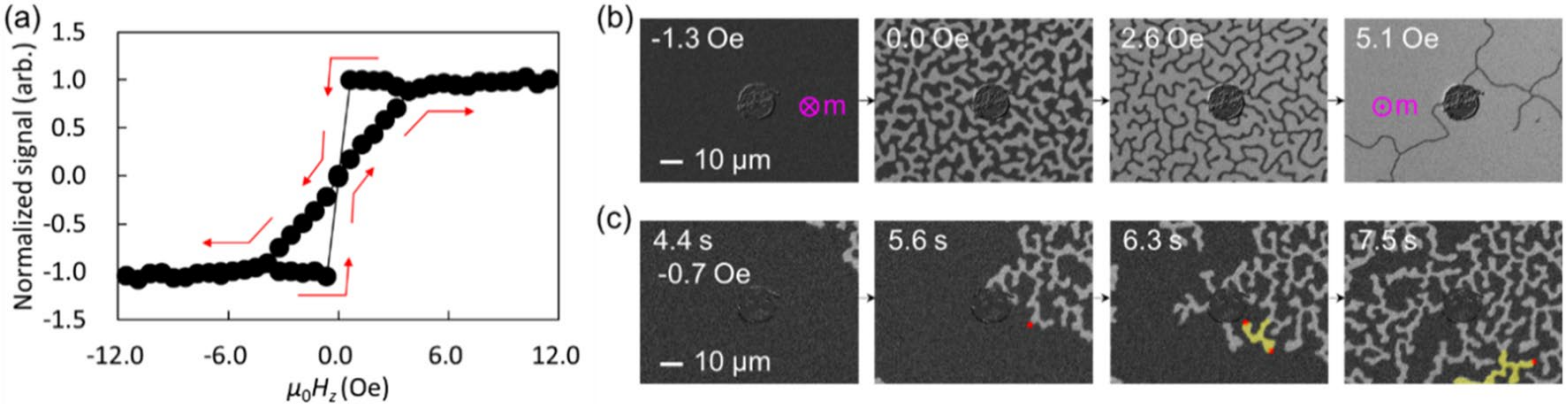
Evolution of magnetization state under perpendicular magnetic field. (**a**) Hysteresis curve obtained from the change in full image brightness. The red arrows indicate the magnetization state following the hysteresis curve as the magnetic field changes. (**b**) Magnetization states observed during increasing magnetic field. (**c**) Stripe formation. A magnetic field of -0.7 Oe was applied to the $- z$ uniform state.

The formation process of the stripe state was further investigated without the use of probe contact. Figure 2a plots a normalized hysteresis loop of the sample in terms of the external magnetic field ($H_{z}$) applied in the $z$-direction. The loop was obtained by measuring the overall brightness of the image, and the brightness values were normalized to $\pm 1$ for the uniform $\pm z$ state. When a sufficient field is applied, the magnetization state becomes a uniform state ($+ z$ or $- z$). At around zero field, the stripe states are observed. Figure 2b demonstrates how the initially uniform $- z$ state changes as the magnetic field is gradually increased. As the magnetic field increases from − 1.3 to 0.0 Oe, the stripe state forms immediately from the $- z$ state, and the signal at the loop becomes nearly zero. Increasing the field to 2.6 Oe widens the area of the $+ z$ region and reduces the $- z$ area because a positive field prefers + z magnetization due to the Zeeman energy. The overall shape of the stripe is maintained despite changes in the relative proportion of $+ z$ and $- z$ areas. However, when the field becomes much larger (5.1 Oe), the thinned $- z$ region disappears one by one, and then changes to a uniform $+ z$ state (not shown here).

Figure 2c provides a visualization of the formation of the stripe state over time. The sample was initially in a uniform $- z$ state at a magnetic field of − 10 Oe and the field was then changed to − 0.7 Oe. Images were captured after changing the field. At 4.4 s, a $+ z$ stripe magnetization appears in the upper right corner of the observation area, which then grows like a maze. The red dots in Fig. 2c denote the stripe ends, while the yellow regions represent newly formed $+ z$ regions from the stripe end. Despite the external magnetic field (− 0.7 Oe) favoring the $- z$ magnetization direction, the $+ z$ magnetization state is created, indicating the presence of an effective magnetic field at the stripe end that is greater than the external field. This effective magnetic field, referred to as $H_{{{\text{stripe}}}}$, acts on the boundary between the uniform and stripe regions and always tries to extend the stripe region. In Fig. 2c, the direction of $H_{{{\text{stripe}}}}$ is in the $+ z$ direction, and its magnitude would be greater than 0.7 Oe.

A brief summary of the sample's properties for magnetic fields follows: near zero magnetic field, the stripe state is stable and dominates the magnetization state. As the external magnetic field is increased, the stripe regions shrink and the uniform magnetization state becomes more stable. When the magnetic field is large enough ($\left| {\mu_{0} H_{z} } \right|$ $\gtrsim$ 4 Oe), the uniform state is completely dominant. The stripe state is created through the growth of stripe ends, driven by an effective magnetic field $H_{{{\text{stripe}}}}$, which acts to extend the stripe regions. Therefore, $H_{{{\text{stripe}}}}$ can be considered the driving force behind the formation of the stripe state.

**Figure 3:**
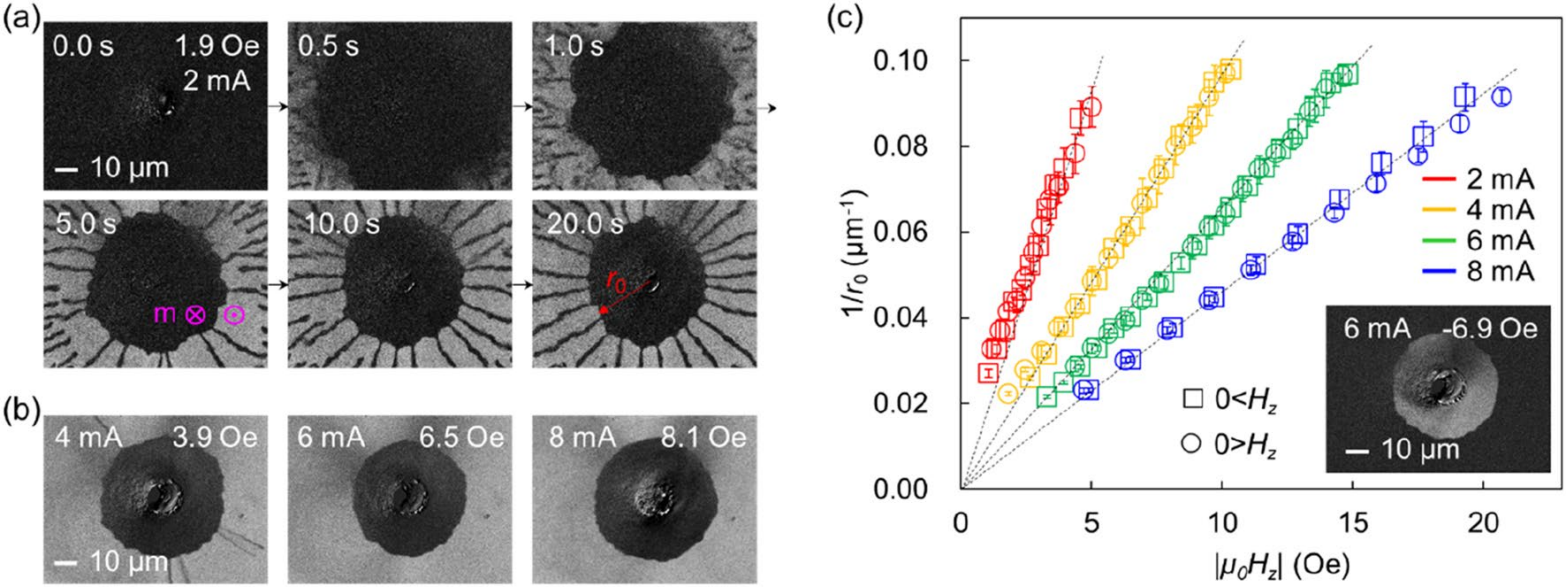
Effect of radial current. (**a**) Formation of a stable circular magnetization state. The red arrows indicate the direction of the current flow and the pink arrows indicate the direction of magnetization. The current is maintained at 2 mA and the magnetic field is changed from -10 Oe to 1.9 Oe at time 0 s. (**b**) Stable magnetization states under various currents and magnetic fields. (**c**) Results of stable radius value measurement as a function of the applied magnetic field and current. The error bars represent the standard deviation.

In the presence of a radial current, the magnetization state of the sample changes. Figure 3a shows what is observed when the external field is changed from − 10 to 1.9 Oe with a constant current of 2 mA. After 0.5 s, $+ z$ stripes generated outside the observation area are pushed into the image by the positive $H_{z}$ and $H_{{{\text{stripe}}}}$. The generation of these stripes is faster than the image capture speed, making the image blurry. After 5 s, the magnetization state change almost stops, and after 10 s, a stationary magnetization state is observed. In the stable image at 20 s, it is possible to measure the radius ($r_{0}$) at which the $+ z$ stripe approaches only a certain distance from the center of the inner pad (Fig. 3a). Clearer circular magnetization states can be created by increasing the current and $H_{z}$ values (Fig. 3b and Supplementary Fig. 1). These circular states cannot be observed when only the external magnetic field is applied (as shown in Fig. 2), and are therefore of interest. For reference, if the current and magnetic field are not sufficient, a state where the boundary of the circle is unclear is formed (Supplementary Fig. 2).

Figure 3c shows the relationship between $r_{0}$ and $H_{z}$ for different current values. Note that, experiments were also performed with the initial state being a uniform $+ z$ state and $H_{z}$ being negative and it shows the same tendency regardless of magnetization direction and magnetic field direction. A representative image is shown in the inset. If we take all $1/r_{0}$ values and plot them as a function of |$H_{z}$|, we can see a straight-line trend passing through the origin.

This tendency is interpreted as the fact that the effective magnetic field generated by the current is proportional to the magnitude of the current density. The current generates an effective magnetic field in the magnetic material due to the SOT, which can be called $H_{{{\text{SOT}}}}$. The amount of spin injected from the W layer into the CoFeB layer will be proportional to the current density. Thus, the magnitude of $H_{{{\text{SOT}}}}$ can be written as $\mu_{0} H_{{{\text{SOT}}}} = s{ }\varepsilon \left| {j_{{\text{W}}} } \right|{ }\left[ {\text{T}} \right]$. Here, $j_{{\text{W}}}$ is the current density in the W layers with a unit of A/m^2^ and $\varepsilon$ is an efficiency of the SOT with a unit of T m^2^/A. $s$ ($= + 1$ or $-$ 1) is a sign determined by both directions of the current and the magnetization change in position. For example, if the direction of magnetization changes from $\pm z$ to $\mp z$ when following the direction of current, *s* is $\pm 1$.

Assuming that the current flows only in the conducting layers (W and CoFeB) and the layers are so thin that the current density of the two layers is the same, $j_{{\text{W}}}$ will be $I/\left( {2\pi rd_{{\text{c}}} } \right)$, where $I$ is the total current and $d_{{\text{c}}}$ is the total thickness of the conducting layers (4.3 nm). For the stripe end to remain stationary, the sum of all the magnetic fields must be zero. Expressing this as a formula, $H_{{{\text{stripe}}}} + H_{z} + H_{{{\text{SOT}}}} = 0$. Ignoring $H_{{{\text{stripe}}}}$, because it is relatively smaller than $H_{z}$, gives an exact linear relationship between $H_{z}$ and $1/r_{0}$.

**Figure 4:**
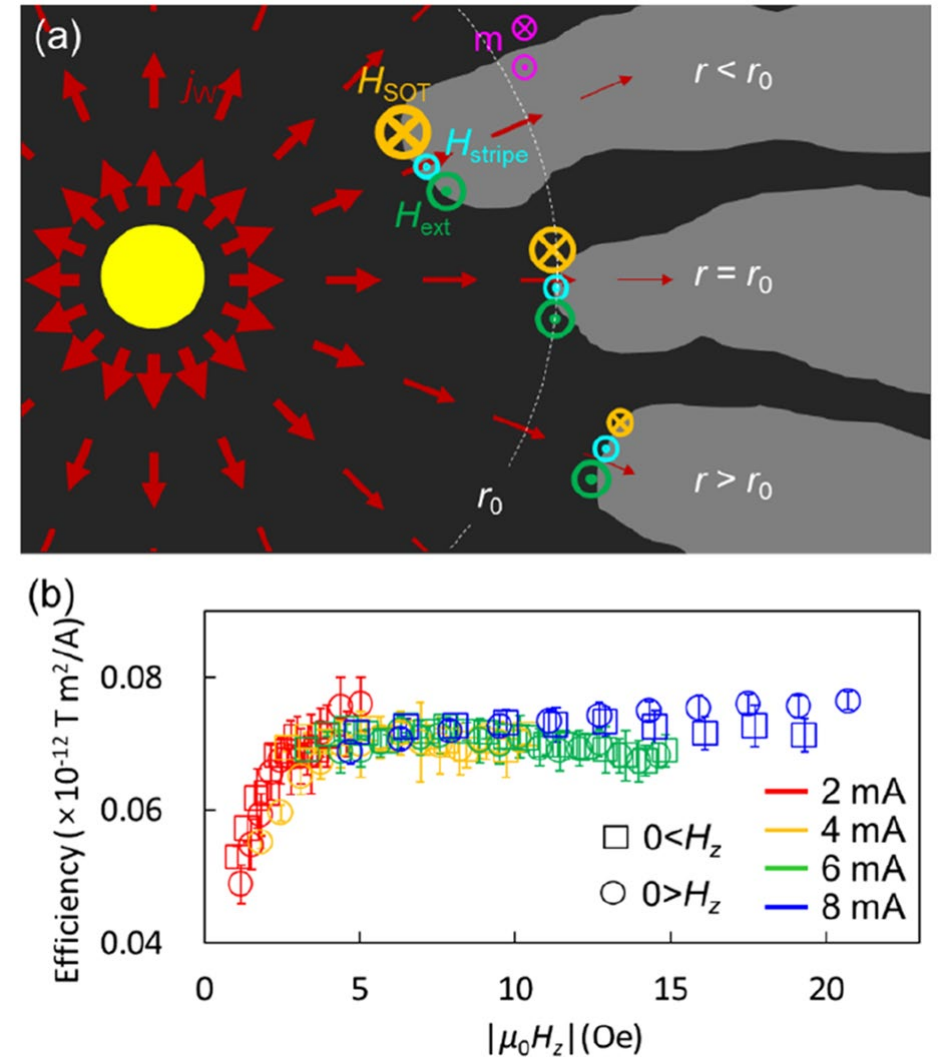
Stability of radius and measurement of the SOT efficiency. (**a**) Schematic illustration of the stability of the radius and $H_{{{\text{SOT}}}}$ on the stripe end position. (**b**) SOT efficiency $\varepsilon$ as a function of applied $H_{z}$ with ignoring $H_{{{\text{stripe}}}}$.

In Fig. 4a, the stability of $r_{0}$ is schematically shown. When the stripe end is pushed inward beyond $r_{0}$, it experiences a higher current density, leading to an increase in $\left| {H_{{{\text{SOT}}}} } \right|$. In Fig. 4a, the direction of $H_{{{\text{SOT}}}}$ is $- z$, causing the stripe end with $+ z$ magnetization to be pushed outward. Conversely, as the stripe end moves further away than $r_{0}$, $\left| {H_{{{\text{SOT}}}} } \right|$ decreases and it is unable to cancel out $H_{{{\text{stripe}}}} + H_{z}$. In this case, the direction of $H_{{{\text{stripe}}}} + H_{z}$ is $+ z$, resulting in the growth of the stripe end inward. Due to this restoration effect, the stripe end or the magnetic domain wall exist stably at *r*_0_.

The primary finding of this study is the ability to determine the efficiency of the SOT from the relationship between $H_{z}$ and $r_{0}$. Figure 4b illustrates how $\varepsilon$ ( =$\left| {\mu_{0} H_{z} 2\pi r_{0} d_{{\text{c}}} /I} \right|$) varies with $H_{z}$, with $H_{{{\text{stripe}}}}$ ignored. When $H_{z}$ is sufficiently large, the SOT efficiency converges to a constant value independent of the current. These results suggest that the SOT efficiency can be determined using a single image of a stable magnetization state. However, when the external field is small ($\left| {\mu_{0} H_{z} } \right|$ $\lesssim$ 4 Oe), the measured efficiency is lower because the magnetization state still possesses a stripe structure even when pushed towards the inner pad. Since $H_{{{\text{stripe}}}}$ is non-zero in the presence of a stripe structure, eliminating the stripe state is necessary to increase the accuracy of SOT efficiency measurements using the radial current geometry. Note that, we performed a comparison with a method that measures magnetic domain wall movement speed^42,43^, and similar results were obtained (Supplementary Note 1).

This ability to measure SOT from a single image means that the proposed method is easier to apply than other methods. This ease of use includes the fact that there is no need for complex patterning of the magnetic film and no strong magnetic field is needed to tilt the magnetization vector away from the magnetic anisotropy axis.

Despite these advantages, this measurement method has several limitations. First of all, we need well-made PMA films. PMA films with poor uniformity, nucleation occurs more easily than magnetic domain wall motion. In a specific situation, if nucleation occurs inside the circular magnetic domain while the circular domain is being formed, the circular domain disappears. Therefore, samples with few defects are required.

The domain wall of a well-made PMA film is easily moved by a perpendicular magnetic field, but does not move easily by an in-plane magnetic field. This also means that only the damping-like component of the SOT effect is measured. Due to the spin Hall effect^11,12,35,37^, the current flowing through the W layer causes spin-polarized electrons to accumulate on the surface. These accumulated spins are injected into the CoFeB layer, which is called spin pumping. The pumped spin induces damping-like SOT and field-like SOT^22^. Both SOTs act as effective magnetic fields. The effective field induced by the damping-like torque is proportional to $\left| {j_{{\text{W}}} } \right|{{\varvec{\upsigma}}} \times {\mathbf{m}}$. The field-like torque generates a magnetic field proportional to $\left| {j_{{\text{W}}} } \right|{{\varvec{\upsigma}}}$. Here, ${{\varvec{\upsigma}}}$ is the pumped spin direction. The direction of ${{\varvec{\upsigma}}}$ is determined to be perpendicular to both the direction of current and the direction of pumping. As a result, the field-like SOT acts like an in-plane magnetic field. Since the in-plane magnetic field cannot move the magnetic domain walls of the PMA sample, it is not suitable for measuring the field-like SOT. In contrast, the damping-like SOT can induce an effective perpendicular magnetic field to the magnetic domain wall. This is because at the center of the magnetic domain wall, the wall must have a pure in-plane magnetization component, and if this in-plane direction is perpendicular to ${{\varvec{\upsigma}}}$, the effective magnetic field becomes perpendicular. It is notable that $\varepsilon$ varies depending on whether the DW structure is Néel type or Bloch type^35,43^, but this was not considered in this paper.

## Conclusion

This study has identified unique magnetization states that arise from the interplay between the external magnetic field and the radial current. These unusual magnetization states form a large circular region with a $\pm z$ magnetization that is typically difficult to observe in samples that favor the stripe state. This phenomenon arises from the cancellation of several magnetic fields, and the effective magnetic field generated by the current has a characteristic that is proportional to the inverse of the radius. Consequently, all magnetization changes stop at a specific radius, making it possible to measure the efficiency of the current in generating an effective magnetic field with a single image. This discovery has significant implications for the field of spintronics and may serve as a starting point for further research on non-uniform current.

### Supplementary Information

Supplementary Information.

## Data Availability

The datasets generated and/or analyzed during the current study are available from the corresponding author upon reasonable request.
