# Supplementary material for: Measurement of spin–orbit torque using field counterbalancing in radial current geometry
Source: Sci Rep. 2023 Nov 8;13:19357. doi: 10.1038/s41598-023-46658-z (PMC10632434; doi:10.1038/s41598-023-46658-z)
Supplement: Supplementary file 1 — Supplementary Information. [file 41598_2023_46658_MOESM1_ESM.docx]

Supplementary Information

**Measurement of spin-orbit torque using field counterbalancing in radial current geometry**

Jong Wan Son, Seungmo Yang, Tae-Seong Ju, Chanyong Hwang, and Kyoung-Woong Moon

*Quantum Spin Team, Korea Research Institute of Standards and Science, Daejeon, 34113, Republic of Korea*

*Correspondence to be addressed to: cyhwang@kriss.re.kr (C. Hwang), kwmoon@kriss.re.kr (K.-W. Moon)

This file contains:

Supplementary Figure 1-3

Supplementary Note 1

Supplementary References


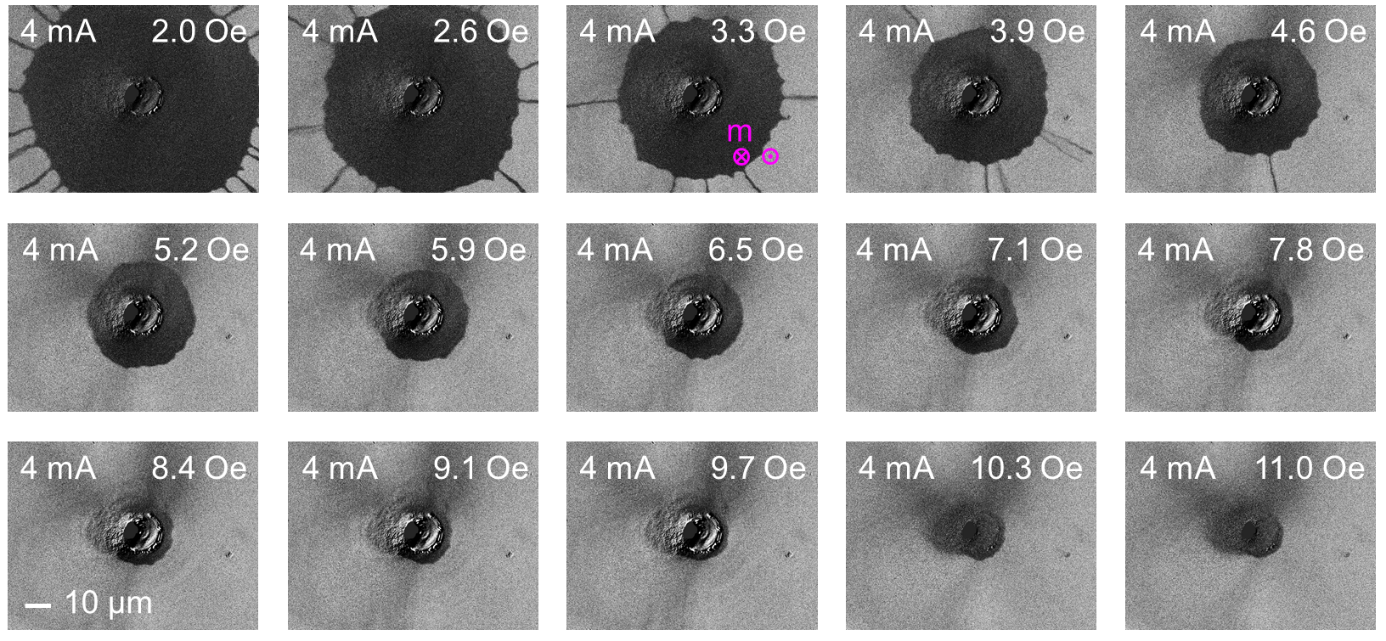


**Supplementary Figure 1.** Magnetization states that are stabilized according to the applied perpendicular magnetic field while the current is fixed at 4.0 mA. These are images with the background image removed.


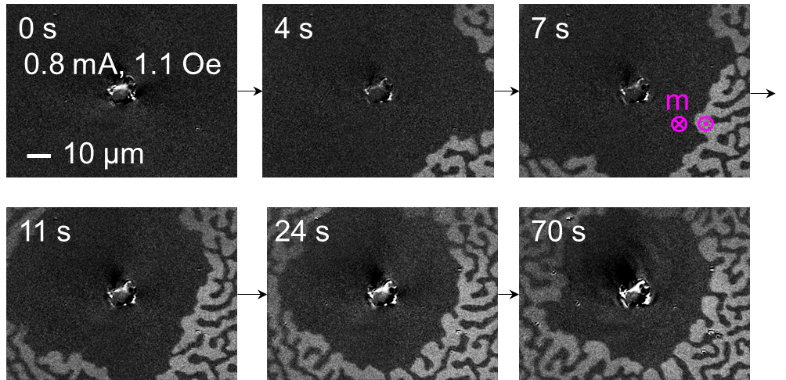


**Supplementary Figure 2.** Formation of an incomplete circular magnetic domain by weak current and weak magnetic field. A current of 0.8 mA and a perpendicular magnetic field of 1.1 Oe are applied to the initial down state. Over time, the up domain grows, but radial currents prevent it from growing to the center of the image. These are images with the background image removed.

**Supplementary Note 1**

To verify the validity of the proposed measurement method, a previously known measurement method was performed. The method is to measure the speed of the domain wall moving by a magnetic field in a wire-structured sample while passing a weak current^1,2^. The sample was produced by patterning the layered structure used in the text to a width of 10 μm and then depositing gold electrodes (Supplementary Fig. 3(a)). The wire is thin enough that magnetic domain walls with well-defined positions can exist. Supplementary Figure 3(b) shows the domain wall speed as a function of the perpendicular magnetic field ($H_{z}$) with additional current. Here, the positive current helps the magnetic domain wall move, and the negative current hinders the magnetic domain wall movement. Converting the effect of the current into an effective magnetic field like $\varepsilon j_{W}$ and redraw the graph, all results will be combined into one curve regardless of the current density (Supplementary Fig. 3(c)). Here, $j_{W}$ is the current density in the W layers with a unit of A/m^2^ and $\varepsilon$ is an efficiency of the SOT with a unit of T m^2^/A. The value of $\varepsilon$ measured using this method is 6.2×10^-14^ T m^2^/A, which is similar to the convergence value in Figure 4(b).


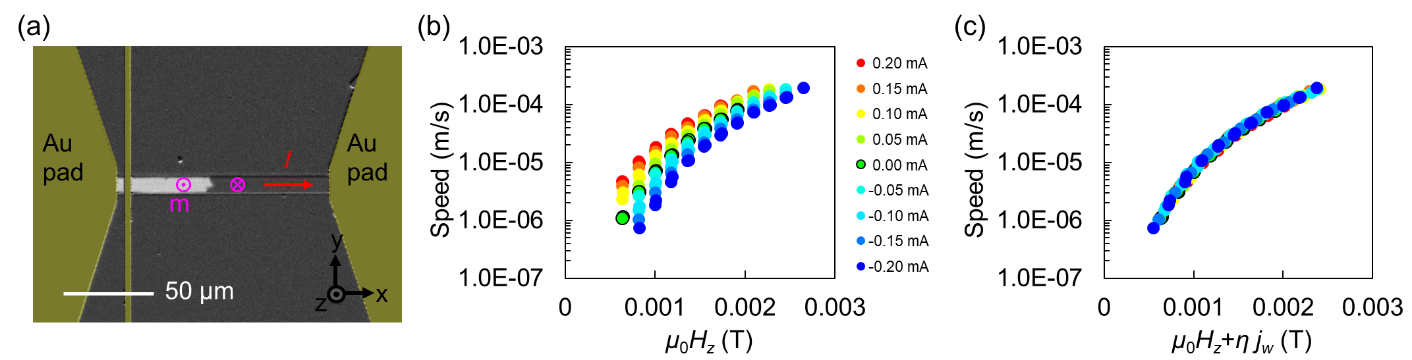


**Supplementary Figure 3.** SOT measurement using magnetic domain wall movement. **(a)** Sample image with background removed. One magnetic domain wall is on the wire with a width of 10 μm. **(b)** The speed of the domain wall moving by the magnetic field measured while applying currents of various magnitudes. The names of the indices represent current values. **(c)** Result of correction by converting the current effect into effective magnetic field.

**Supplementary References**

1. Lee, J.-C. *et al.* Universality classes of magnetic domain wall motion. *Phys. Rev. Lett.* **107**, 067201 (2011).

2. Kim, J.-S. *et al.* Comparison between spin-orbit torques measured by domain-wall motions and harmonic measurements. *AIP Adv.* **8**, 056009 (2019).
